# Legacy leaks, lasting liabilities: elevating abandoned oil and gas wells in climate change mitigation policy

**DOI:** 10.1093/nsr/nwaf249

**Published:** 2025-06-20

**Authors:** Lena Höglund-Isaksson

**Affiliations:** Pollution Management Group, Energy, Climate and Environment Program, International Institute for Applied Systems Analysis (IIASA), Austria

As the world intensifies its efforts to reduce greenhouse gas emissions, targeting methane emissions has emerged with heightened urgency. Methane's atmospheric lifetime is short, but its global warming potential over 20 years is >80 times that of carbon dioxide [1]. Reducing methane emissions is therefore among the most effective strategies for slowing near-term global warming. Among methane sources, one has remained especially elusive: abandoned oil and gas (AOG) wells. These wells, drilled over decades of fossil fuel extraction, often continue leaking methane long after production ends. Many are improperly sealed, unmonitored, and undocumented in official inventories. Some are ‘orphaned’, meaning no legally responsible operator exists. Their emissions are diffuse but persistent, representing a methane source in need of close monitoring and control long after the world has moved away from oil and gas dependency.

AOG wells are not only a methane problem. Unplugged or degraded wells pose serious health threats to local communities as they contaminate aquifers, release toxic volatile organic compounds and—under certain pressure conditions—even pose explosion hazards [2]. They also threaten the long-term viability of carbon capture and storage (CCS) in affected formations, where leakage through legacy wellbores can undermine containment integrity [3].

IPCC's Sixth Assessment Report Working Group III [4] acknowledges methane from AOG wells as a source of uncertainty, but offers no global emission estimate. Starting in 2024, the newly revised reporting framework to the UNFCCC asks Annex-1 countries to report AOG emissions as a separate category [5]. Australia, Canada, the UK and the USA adhered to this call and reported for year 2020 a total 0.32 Mt methane from the source, whereof 0.30 Mt was from the USA alone. There is accordingly a striking lack of completeness in the reporting of these emissions at the global and national scales, including for relatively prosperous countries like Norway and Romania.

The study by Lei *et al.* [6] represents a major step forward in our understanding of the scale of the abandoned wells problem, and opens up for including it as a measurable component of the global methane budget. The authors develop an unparallelled harmonized global inventory of methane emissions from AOG wells, covering 4.5 million wells in 127 countries, and providing a first global benchmark: 0.4 Mt of methane released from abandoned wells in 2022. Although modest in size, they represent <1% of global oil and gas systems methane emissions, their persistency and lack of legal ownership make them important to monitor and quantify. Lei *et al.*’s dataset, CEADs-AOGI, includes well-level information for >420 000 sites, and also covers well attributes relevant for the development of effective mitigation strategies, such as resource type, terrain, plugging and ownership status. By incorporating these into a process-based bottom-up emissions model, the authors are able to reconstruct historical site-level emissions and produce scenario-based projections of future trajectories. Lei *et al.* also introduce a novel corporate attribution analysis, linking methane emissions to historical operators. In several countries, a small number of multinational firms are associated with a disproportionate share of legacy emissions. This raises the possibility of retrospective accountability frameworks, where past emitters could be held financially responsible for cleanup. Such an approach could support just transition strategies, particularly in resource-limited settings where national governments inherit the burden of remediation.

A striking finding is the presence of ‘super-emitters’ and the high degree of intermittency and spatial variability in methane emission rates across AOG wells. Authors highlight that this is not only a problem for onshore wells; shallow offshore wells like in the North Sea can exhibit huge emission rates due to challenging decommissioning conditions and aging legacy infrastructure. This calls for frequent monitoring of emissions from abandoned wells, e.g. using satellite remote sensing technology. Like for so many other methane source sectors, effective mitigation strategies must combine frequent monitoring of emissions with site-specific bottom-up information on the geological context, technological set-up, and ownership structures, i.e. the kind of information compiled and presented here by Lei *et al.* in great detail for AOG wells. It's when site-specific bottom-up information is linked to top-down emissions quantifications that regulators have the tools to jump-start strong and legally binding methane mitigation efforts.
